# Beyond Lassa Fever: Systemic and structural barriers to disease detection and response in Sierra Leone

**DOI:** 10.1371/journal.pntd.0010423

**Published:** 2022-05-19

**Authors:** Hana Rohan

**Affiliations:** UK-Public Health Rapid Support Team and London School of Hygiene and Tropical Medicine, London, United Kingdom; NIAID Integrated Research Facility, UNITED STATES

## Abstract

**Background:**

Lassa fever (LF) often presents clinically as undifferentiated febrile illness. Lassa Fever cases in Sierra Leone have been falling since the 2014–2016 Ebola epidemic. Data from other LF endemic countries suggest that this is not a true reflection of local epidemiological decline, but rather a function of either health seeking behaviour or the health/referral system. In Sierra Leone, many other diseases present with a similar early clinical picture, including COVID-19 and Marburg Disease (which has recently emerged in neighbouring Guinea). This empirical study explores the implementation of health system processes associated with International Health Regulations (IHR) requirements for early detection and timely and effective responses to the spread of febrile disease, through the case study of LF in Sierra Leone.

**Methodology/Principal findings:**

This study used a qualitative approach to analyse local policy and guidance documents, key informant interviews with policy and practice actors, and focus group discussions and in-depth interviews with health care workers (HCWs) and community health workers (CHWs) in Kenema District to examine the ways in which undifferentiated fever surveillance and response policies and processes were implemented in the post-Ebola period. Multiple challenges were identified, including: issues with the LF case definition, approaches to differential diagnosis, specimen transport and the provision of results, and ownership of laboratory data. These issues lead to delays in diagnosis, and potentially worse outcomes for individual patients, as well as affecting the system’s ability to respond to outbreak-prone disease.

**Conclusions/Significance:**

Identification of ways to improve the system requires balancing vertical disease surveillance programmes against other population health needs. Therefore, health system challenges to early identification of LF specifically have implications for the effectiveness of the wider Integrated Disease Surveillance and Response (IDSR) system in Sierra Leone more generally. Sentinel surveillance or improved surveillance at maternity facilities would help improve viral haemorrhagic fever (VHF) surveillance, as well as knowledge of LF epidemiology. Strengthening surveillance for vertical disease programmes, if correctly targeted, could have downstream benefits for COVID-19 surveillance and response as well as the wider health system—and therefore patient outcomes more generally.

## Author summary

Lassa fever (LF) often presents clinically as undifferentiated febrile illness. Lassa Fever cases in Sierra Leone have been falling since the 2014–2016 Ebola epidemic. Data from other LF-endemic countries suggest the drop in case numbers reflects reduced health seeking behaviour or issues within the surveillance, response and health systems. In Sierra Leone, many other diseases present with a similar early clinical picture, including COVID-19, meaning that findings from a case study of LF have wider applicability. There are no recent empirical studies of the functionality of Sierra Leone’s disease surveillance and response system. Qualitative analysis of policy documents and primary data collected from within the health system identified multiple challenges including: issues with the LF case definition, approaches to differential diagnosis, specimen transport and the provision of results, and ownership of laboratory data. These issues lead to delays in diagnosis, and potentially worse outcomes for individual patients, as well as affecting the system’s ability to respond to outbreak-prone disease.

## Introduction

In Sierra Leone, Lassa fever (LF) cases have been falling since the 2014–2016 Ebola epidemic, which alongside increased duration between illness onset and presentation, suggests presentation at later stages of illness [1,2]. Further, data from other endemic countries [3,4] suggest that these declines may not be a true reflection of the local epidemiology, but rather a function of either health seeking behaviour or the health/referral system.

This study qualitatively examines health system processes associated with timely identification, diagnosis, and referral of suspected LF, seeks to understand what factors can facilitate or undermine implementation, and explores realistic ways in which the system could be further strengthened.

### Background

Lassa Fever is a rodent-borne Viral Haemorrhagic Fever (VHF) disease that is endemic in West Africa with 300,000 cases estimated annually across the region [5]. Sierra Leone is considered to have the greatest LF incidence globally [6]; Nigeria, Guinea, and Liberia are also LF-endemic countries [7]. While LF is endemic in the Mano River countries (Sierra Leone, Guinea, Liberia, and Cote D’Ivoire) and therefore cases can occur at any time of year, they are primarily driven by interactions between LF’s rat reservoir population, *Mastomys natalensis*, and humans, with human-to-human transmission estimated to account for approximately 20% of cases [8]. Evidence increasingly suggests that rainfall and food availability may affect both the population of *M*. *natalensis* and its interaction with humans [9], and so LF case numbers can rise and fall with the seasons [10,11].

Lassa Fever has an incubation period of 5–16 days [6], and symptoms usually have a gradual onset, with fever, headache and malaise [12] graduating to symptoms like abdominal pain and mucosal bleeding, and more in severe cases [7,13]. There is some debate over the case fatality rate from LF, with estimates among *hospitalised* cases ranging from 1 to 69% [7,14,15], however demographics, comorbidities, and pregnancy status can negatively affect outcomes [14,16]. Most LF cases are mild or subclinical [9]. The absence of population-based studies and challenges with identification of milder cases likely increase estimates of overall mortality from the disease. The provision of supportive care and the antiviral ribavarin are the most common treatment options for LF, and treatment is most efficacious when administered within the first 6 days of illness [5]; early diagnosis, referral, and health-seeking are therefore critical for positive outcomes. Lassa Fever confirmation in Sierra Leone currently utilises PCR tests [17], although early data suggest good sensitivity and specificity for a rapid diagnostic test (RDT) being developed by a team at Tulane University [18].

Lassa Fever, like Ebola, is a VHF that has been identified by the World Health Organisation (WHO) as a “severe emerging disease with potential to generate a public health emergency, and for which no, or insufficient, preventive and curative solutions exist(s)” [19] and therefore as a priority pathogen for research and development. However, given the estimated proportion of cases attributable to human-to-human transmission, it is not clear how likely LF is to spark large, generalised outbreaks that could meet the threshold for a public health emergency. While larger outbreaks have occurred in recent years in Nigeria [4,20], alongside uncertainty about the extent to which improved surveillance may be contributing, it is also unclear how much these have been driven by changes in climate and therefore rodent ecology, rather than by human-to-human transmission.

### Lassa Fever in Sierra Leone

**Fig 1:**
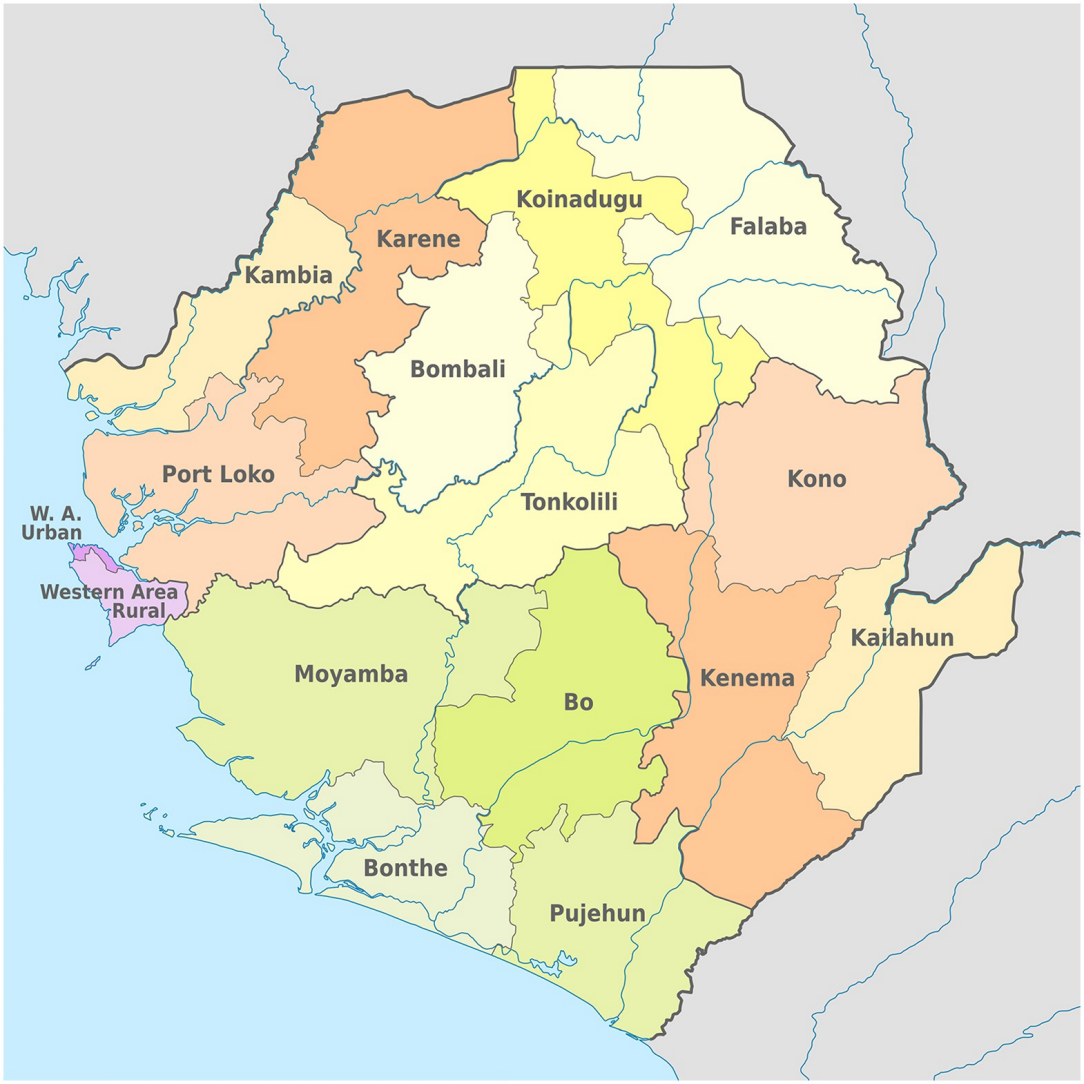
Map of Sierra Leone with District Boundaries. Map of Sierra Leone showing District and Chiefdom Borders. Map source: https://commons.wikimedia.org/wiki/File:Districts_in_Sierra_Leone_2018.svg.

Sierra Leone is made up of 16 districts; LF has traditionally been associated with the districts of Kenema, Bo, Kono, and Kailahun, in the south-east of the country [21,22] (see Fig 1), although some data suggest it may be endemic throughout Sierra Leone, with the historical emphasis on LF prevalence in the south-east reflecting more focused surveillance there, rather than an accurate geographical picture of disease burden [23,24].

In Sierra Leone, the Kenema Government Hospital (KGH) houses a national VHF laboratory that was the only facility able to test for LF both prior to and during the Ebola outbreak. It grew out of the historical LF laboratory, a regional reference laboratory for LF in West Africa [25,26], and to date is still the only laboratory in the country authorised to officially confirm a LF diagnosis [27]. The KGH site is also the only site providing clinical care, community outreach and rodent control activities for LF treatment and prevention within Kenema District [21,28]. At the start of the Ebola outbreak, a number of different foreign research interests were associated with the LF team at KGH, including Tulane University (who were members of a US-led VHF Consortium with long-term research interests in Kenema), USAMRIID (US Army Medical Research Institute of Infectious Diseases), and private research organisations like Metabiota [21,29]. The enduring presence of these foreign groups in Kenema has had implications for the structure of local health and surveillance systems, with a global health security agenda dominating research interests and often health system resourcing decisions [21].

Alongside LF, Sierra Leone has a high burden of diseases that have a very similar initial clinical presentation [22], such as malaria, typhoid, influenzas, and now COVID-19. The 2016 Malaria Indicator Survey found that 52% of children tested RDT positive for malaria [30], and a study of febrile patients at KGH showed that among those diagnosed with malaria and those discharged with no diagnosis, the five most prevalent symptoms were identical [31].

Of the three countries primarily affected by 2014–2016 Ebola outbreak, Sierra Leone was the worst affected, with over 14,000 confirmed cases and nearly 4,000 deaths [32]. The crisis highlighted the weakness of the health system, and simultaneously had a substantial impact on access to routine care [33] as the health system was almost entirely re-oriented to Ebola response, and trust in it collapsed [34]. As a result of that outbreak, which highlighted inadequate surveillance and response systems [35], much of the focus of donor-sponsored health system rebuilding in the aftermath of the outbreak has been on improvements to surveillance and other International Health Regulations (IHR)-mandated health system components, with health security increasingly dominating the policy discourse in Sierra Leone [36]. While the epidemic drew attention to the health system fragmentation that had contributed to the Ebola epidemic, recovery efforts that prioritised a donor-led health security agenda have contributed to a further fragmentation of the system [36–39].

Both suspected and confirmed LF case numbers have declined substantially in the post-Ebola period relative to the pre-Ebola period, with average annual admitted cases declining from 148 in 2011–2013, to only 38 in 2016–2019 [2]. Recent LF case numbers from other endemic countries such as Nigeria [4] and Liberia [3] have been increasing, suggesting that these admissions data do not reflect a true reduction in cases, and that other factors associated with the health system’s ability to support timely referrals, or health seeking behaviour may be contributing. Health seeking behaviour goes beyond the scope of this study, but will be examined in future manuscripts.

This study therefore explores the implementation of health system processes associated with IHR requirements for early detection, and timely and effective responses to the spread of disease [40] through the case study of LF in Sierra Leone. Through this case study approach, this study seeks to understand the ways that systemic (e.g. standard operating procedures (SOPs), diagnostics, formal health system processes) and structural factors (e.g. Sierra Leone’s economy and therefore health system resources) in the post-Ebola period affect availability of access to infectious disease referral and treatment. Due to the undifferentiated nature of LF and Sierra Leone’s wider disease ecosystem, this paper takes a broader view than LF alone, to understand the ways in which LF referral and treatment protocols may differ from those for other diseases, and to situate these within the context of the health system to which they belong.

## Methods

### Ethics statement

Ethical approval for the study was provided by the London School of Hygiene and Tropical Medicine ethics committee, as well as Sierra Leone’s Ethical and Scientific Review Committee. The MOHS in Sierra Leone and the DHMT in Kenema District were briefed and their consent was provided to carry out the research.

Accepted standards for ethical research applied throughout data collection. Respondents received advance written information about the research objectives and were not compelled to participate. All interviews and FGDs were conditional on written consent being provided. The research took place at times designed to cause minimal disruption to clinical processes.

All communities visited have been anonymised in order to protect respondent confidentiality. No quotations that might identify respondents have been used in reporting. Audio data have been stored on a secure internal server at LSHTM and will be destroyed after three years.

In all research activities, participants were not remunerated for participation; refreshments were provided during FGDs.

### Methods

Data collection for this study consisted of a thematic review of all relevant policy and guidance documents, key informant interviews (KIIs) with health system actors and in-depth interviews (IDIs) with Community Health Workers (CHWs), and focus group discussions (FGDs) with health care workers (HCWs) in Kenema district.

A review was conducted of key Sierra Leone health policies, guidance documents and SOPs, Ministry of Health and Sanitation (MOHS) and KGH documents. These were identified through web searches for the terms “Sierra Leone” OR “Kenema” OR “KGH” AND “surveillance” OR “identification” OR “diagnostics” OR “confirmation” OR “lab*” OR “specimen” OR “supply chain” OR “response” OR “outreach” OR “case definition” OR “case management” OR “IPC” OR “infection prevention” OR “PPE”. Identified documents were then manually searched for relevance and reference to additional items. Finally, the author’s professional network and key informants were consulted to compile an exhaustive list.

Several policy and guidance documents lay the groundwork for Sierra Leone’s surveillance and response system, including the Integrated Disease Surveillance and Response (IDSR) guidelines, Specimen Referral Guidelines, the National CHW Policy, alongside several others [17,27,38,41–49] (twelve in total, see S1 Table). Almost all of these were developed either towards the tail end of, or in the aftermath of the Ebola epidemic by the Sierra Leone MOHS, and with support from international partners such as WHO, Public Health England (PHE), and US CDC.

Taken together, the identified documents describe processes for best practice case definition and identification, confirmation, and referral of LF cases. Documents were read and re-read by HR, and coded to a framework based on the core activities outlined by the WHO as necessary for a functional IDSR system [50]. This paper examines those processes through analysis of those documents and compares their content to accounts from health policy actors, HCWs, and CHWS, to explore the implications of those processes and their implementation for surveillance, response, and patient care and outcomes.

Unstructured interviews were conducted with nine MOHS, District Health Management Team (DHMT), and non-governmental organisation (NGO) key informants working at the national and district levels, to understand the systemic and structural factors that may affect implementation and perceptions of those policies and guidance documents. Topic guides varied between key informants according to their area of specialism (e.g. supply chain, clinical management of LF, laboratory processes). Key informants were identified through document review and snowball sampling drawing initially from the existing networks of the study’s investigator, both within Sierra Leone and internationally. Interviews took place between May 2019 and March 2020. One key informant provided a detailed case study of an LF outbreak from Tonkolili District. That case study is included here as a ‘deviant case’ [20] to illustrate health system procedures in a district outside the traditional LF belt. That clinical case study was reconstructed from the interview transcript and notes, and cross-checked with the respondent before finalising.

In Kenema District, eight FGDs were held with HCWs of groups sized between 5 and 8, and semi-structured IDIs were conducted with CHWs at the same sites within Kenema District (2 IDIs per site, 8 in total). Data collection took place between June and October 2019.

Within Kenema District, the sites for this study were purposively selected according to the presence or absence of historical experiences with LF or Ebola cases e.g. two FGDs and two IDIs were held each in chiefdoms with either experiences of both LF and Ebola, neither LF nor Ebola, Ebola and no LF, or LF but no experience of the Ebola epidemic (see Table 1 for distribution of FGDs and IDIs).

**Table 1:** Data collection by site type (experiences of LF and Ebola cases).

	Ebola +	Ebola -
Lassa Fever +	2 x FGDs with HCWs 4 x IDIs with CHWs	2 x FGDs with HCWs 4 x IDIs with CHWs
Lassa Fever -	2 x FGDs with HCWs	2 x FGDs with HCWs

The PHUs nearest each of these selected chiefdoms were identified for recruitment of HCW and CHW respondents. This approach to site selection helped ensure that data reflected a range of HCW/CHW experience and exposure to outbreak-prone diseases in Sierra Leone. These communities were identified by scrutinising data on both LF and Ebola epidemiology, and in collaboration with the LF outreach team at KGH. HCWs were identified through their associated peripheral health units (PHUs) in those communities.

All participants were provided with the study information sheet and informed consent was provided prior to interviews/FGDs. Interviews were conducted in English, Krio or Mende, according to participants’ preferences and recorded on encrypted audio devices. Data were translated and transcribed using an external agency, with spot checks and back translations conducted on a sub-sample of transcripts to confirm translation/transcription quality.

Health Care Worker and CHW data were analysed in NVivo 12 using an inductive approach to ensure that HCW/CHW lived experiences of health care provision were captured. Key informant data were analysed in NVivo 12 using both inductive and deductive approaches, with topic guides acting as a coding framework. Topic guides for interviews conducted later on in the fieldwork schedule were guided by the themes that emerged in earlier analyses, and these also informed the sampling of key informants by helping to identify gaps in the emerging thematic framework and achieve thematic saturation [51]. Topic guides for all respondent groups were piloted prior to data collection and refined in collaboration with local research assistants and the KGH LF outreach team.

## Results

This study identifies multiple systemic and structural factors leading to delays in diagnosis, and potentially worse outcomes for individual patients, as well as the capacity of the system to respond to outbreak-prone diseases. These include: challenges with the LF case definition, approaches to differential diagnosis, specimen transport and the provision of results, and ownership of laboratory data, with consequences for patient management and outcomes. These multiple challenges are further explored through a case study of an LF outbreak in Tonkolili district, outside of the traditional LF belt. Identifying ways to circumvent these challenges with the core activities (comparable to the WHO’s health system ‘building blocks’ [52]) within Sierra Leone’s surveillance and response system has implications for surveillance risk thresholds, and the tensions between vertical programme delivery and horizontal health systems strengthening.

### Case definition challenges

In Sierra Leone, the IDSR Guidelines specify that the LF suspect case definition falls under two different categories: a) acute VHFs, and b) suspect LF or Crimean Congo Haemorrhagic fevers (CCHF). Sierra Leone has reported one case of CCHF between 1956 and 2020 [53]. For the former, acute onset of high fever and any two acute haemorrhagic symptoms (e.g. haemorrhagic or purpuric rash, bleeding nose, blood in stool, etc) should trigger suspicion of an acute VHF. However, given that such haemorrhagic symptoms would reflect already severe disease, relying solely on the acute case definition would likely miss both severe cases in their early stages, and potentially large numbers of milder, but potentially still infectious cases. The suspect case definition for (non-severe or ‘acute’) LF should therefore enable identification of cases earlier in the disease life-course in order to reduce missed cases and to help improve patient outcomes. It specifies “illness with gradual onset with one or more of the following: malaise, fever, headache, sore throat, cough, nausea, vomiting, diarrhoea, myalgia, chest pain, hearing loss **and a history of contact with excreta of rodents or with a case of Lassa Fever**” ([27] p. 45, bolding author’s own). There are two challenges with implementing this case definition: 1) the constellation of symptoms resembles those of almost all febrile illnesses in Sierra Leone and is therefore highly non-specific; and 2) the requirement to know a patient’s history of contact with rodent excreta or LF patients to meet the case definition, which might be challenging for staff at PHUs to confirm, as well as for patients who may not realise they have been exposed.

Some health workers based at PHUs in Kenema District discussed the challenges posed by the two different approaches to LF case definition, as well as the non-specific nature of almost all diseases that they might encounter, and noted the importance of considering a LF diagnosis when a patient presents with undifferentiated febrile illness:

*“So Lassa fever*, *typhoid*, *malaria*, *fever they all have similar signs and symptoms*, *that is why if you are at the centre*, *be careful*, *we have case definition of the minor side and the severe ones for some signs that we were seeing and did not even know that they were Lassa fever signs”*

(HCW, Kenema District, Lassa+/Ebola- chiefdom)

In practice, LF suspicions are raised in Kenema District when fever and other non-specific febrile symptoms persist despite treatment for malaria, and/or when administered RDTs for malaria produce a negative result:

*“Usually at community level*, *Lassa has similar signs and symptoms with malaria with an on and off fever*. *So*, *when it is treated for malaria*, *and they don’t respond to treatment those are the first queries*. *So*, *they know this patient isn’t responding to antimalarials*, *this patient is not responding to antibiotic*, *then they need to do further investigations”*

(MOHS surveillance staff member, Kenema District).

It is well documented that these non-specific symptoms make clinical diagnosis of LF particularly challenging, particularly in early stages of the disease, when treatment with ribavirin might still be beneficial [54]. Additionally, patients in Kenema sometimes present with one or more co-infections, and therefore can test positive for malaria while also being infected with LF or other common febrile illnesses. As one CHW noted, *“Malaria… is too much here*. *It is only [a] few people that you would test for malaria*, *and they don’t have it*, *but it is not that easy*. *It is not even up to 5% of people you can test without malaria*.” (Community Health Worker, Kenema District, Lassa+/Ebola+ chiefdom). Malaria prevalence (among children) in Kenema has been estimated at 38% [30], suggesting that the chances of co-infection are high, and studies have shown that in Lassa endemic areas, LF/malaria co-infection may range from 30–56% [55,56]. Health care worker perceptions of an even higher burden of malarial disease influences clinical decision-making, with HCWs typically administering malaria treatment, and then only re-engaging with a patient’s care after the three-day course, if fever and/or other symptoms persist:

*“Malaria treatment usually last[s] for three days and if the person or child continues to have fever it means there is another cause*. *We usually tell them to bring the child after three days or person back for check-up according to what I was saying*, *I treated a girl for three days*, *but she did not respond to the malaria treatment*, *she came back after three days with signs of Lassa fever”*

(HCW, Kenema District, Lassa+/Ebola+ chiefdom)

### Differential diagnosis

Some health workers reported that if a patient did not respond to malaria treatment, their ‘protocols’ require them to then initiate treatment for typhoid (another relatively common disease in Sierra Leone [57]) and wait for the patient to improve before referring to the district hospital for further investigations. While these diagnostic protocols were reported by health care workers, no written protocol was identified that specified this approach.

*“I will send him to do test if the fever still persists and after an initial visit to me*. *According to our protocol*, *you still need to treat this person for another condition like typhoid*. *If he did not improve after being treated for typhoid again*, *then you have to refer him to the district hospital*.*”*

(HCW, Kenema District, Lassa+/Ebola+ chiefdom)

However, most studies in Sierra Leone, and indeed the health system itself, rely on Widal tests to diagnose typhoid. Widal tests have poor sensitivity and specificity for typhoid [58], making it difficult to establish an understanding of true prevalence; for HCWs relying on Widal, a high positivity rate, whether accurate or not, may lead to an assumption that treating for typhoid is an appropriate approach to differential diagnosis (with attendant consequences for antibiotic use and antimicrobial resistance). Even basic laboratory investigations for these common diseases are not always possible; due to the sheer prevalence of undifferentiated febrile illness in Sierra Leone, supply chain issues, and challenges with specimen transport, meaning that HCWs often have to rely on clinical signs and symptoms:

*“There’s a lot of undifferentiated fever and there’s a lot of fever that receives no diagnostics at all because people just don’t have access to the tests*, *there’s regular stock outs of RDTs and people don’t get blood films in a timely manner”*

(Infectious Disease clinician, iNGO, Freetown).

Taken together, the lack of diagnostic capacity at the PHU level, the undifferentiated nature of most diseases, and the non-specific nature of the suspect LF case definition, many febrile/LF patients could experience long delays before referral to KGH (the only site in the district capable of lab-confirming LF cases) as a result of this process of trying to diagnose by step-wise elimination. The causes and consequences of these delays are well-recognised at the national level:

*“The case fatality for Lassa is high because the PHU staff hold on to this patient for an unnecessarily long period*, *they treated them for one course of malaria*, *another course of malaria and the patient was still not improving and they keep treating for malaria or typhoid”*

(MOHS Surveillance staff, Freetown)

The solution, according to health system key informants, is improved health worker sensitisation on LF, alongside more rapid laboratory confirmation of disease:

*“Any case that comes to the facility you test for malaria*, *it’s negative*, *but the fever is still running*, *don’t try them with a malaria drug*, *send the patient to hospital for testing*. *That’s what they are not doing now*. *They are not doing that*, *even hospitals are not doing that*. *They keep trying antibiotics”*.

(MOHS Surveillance staff, Freetown)

One health worker’s account of their first experience with a LF patient bears this out, despite practicing in the traditional LF ‘belt’, and reaffirms concerns that delays in diagnosing LF can lead to delays in subsequent public health measures, such as follow up and isolation of contacts, and ecological interventions:

*“The first time that I encountered [a] Lassa fever case … I didn’t know anything about it*, *but I was getting news about it because I was residing at the zone*, *so when the patient came*, *we started treating the patient—we were given him antibiotics but the more we were giving him the treatment*, *the more it was getting worse*, *so we had to refer him and later confirmed it was a Lassa fever patient who died*. *We were kept in an isolation centre for some time*, *but thanks to God we did not get the sick”*

(HCW, Kenema district, Ebola-/Lassa+ chiefdom)

At the other end of the case definition spectrum, the acute VHF case definition suffers from similar problems of differentiation: Ebola, LF, and Marburg (recently identified in Sierra Leone, although not in humans [59], and now emerging in human populations in neighbouring Guinea [60]) are all IHR notifiable epidemic-prone VHFs and difficult to distinguish from one another based on the clinical picture alone:

*“At clinical level and community level it will be very difficult to differentiate the initial picture of these symptoms for Lassa*, *for Marburg*, *for Ebola*, *it will be difficult*… *You find it difficult to look at the picture initially of the symptoms to differentiate at that level*, *Marburg*, *Ebola*, *Lassa*, *it’s difficult*. *Extremely difficult*. *The only clarification you have is going to be the lab”*

(MOHS Surveillance staff, Freetown)

Therefore, once a patient is suspected of having LF, laboratory confirmation is critical. The IDSR specifies that laboratory confirmation of disease is “essential” [27], not least because LF, as a VHF, is considered a condition that is potentially notifiable to WHO under the IHR, and the MOHS acknowledges that the laboratory system is not currently able to meet its IHR or GHSA obligations [38]. Given the challenges with distinguishing different acute VHFs from one another clinically, differential diagnosis requires that specimens are laboratory tested for all three VHFs known to have a foothold in Sierra Leone:

*“There is no point in sending a sample for EVD only*, *because it could be… Lassa*. *In actual fact*, *in the context here*, *it’s far more likely … over the next few years*, *that Lassa is going to be there rather than EVD*. *One has to be vigilant for EVD—but it’s the same algorithm pretty much which will identify a high-risk person for a viral haemorrhagic fever of whatever cause*, *and they’ve now added Marburg into that (as they found Marburg)”*

(WHO clinical staff, Freetown).

### Sample transportation and timely results

The Kenema reference laboratory at KGH is the national reference lab for LF; any suspect LF case specimen, from anywhere in the country, should be sent to Kenema for confirmation [17]. According to the draft Specimen Referral Guidelines from 2019 [46], suspect VHF samples (typically LF or Ebola) should be referred to one of the five regional or three tertiary laboratories, although not all were, at the time of data collection, capacitated to run all of the appropriate diagnostics. As a result, suspect cases should “*either [have] two specimens taken or one specimen that’s split*, *so depending on where they are… the Lassa specimen should be sent to Kenema*, *and then the other specimen will be sent to one of the EVD labs which is either Bo*, *Makeni or CPHRL [Central Public Health Reference Laboratory] to go [to] Jui or Connaught Hospital”* (WHO lab staff, Freetown).

This approach necessarily entails specimen transport to two distinct laboratories, often in different districts, with differential diagnosis then dependent on timely reporting from both. Lassa Fever diagnostics are always reported by the Kenema lab, which is the only laboratory authorised to officially confirm LF, although the Sierra Leone-China Bio-Safety Fixed Laboratory (at Jui hospital, near Freetown) and the CPHRL are also identified by Sierra Leone’s Special Pathogen Algorithm [17] as having LF PCR capacity. With Kenema in the east of the country, even when samples were sent directly, suspect LF cases from other districts often experience delays in receiving results, not least because of limitations in the intra-district specimen transport system:

*“I mean most of the districts should have transport and I think WHO might have provided a lot of that during Ebola but*, *you know*, *motorbikes go missing*, *then there’s no fuel*. *It’s fine having a vehicle*, *but if there’s no money for fuel what can you do*?*”*

(WHO laboratory staff, Freetown)

Delays in specimen transport weren’t always related to resource shortages however, but also to the specimen referral system itself—an issue acknowledged in the MOHS’ 2016 National Lab Assessment [41]. Surveillance staff from Kenema District reported that specimen transport to Bo or Freetown (where EVD testing can take place) occurs once a week, on a Thursday. Given the importance of rapid diagnosis for VHFs, systemic delays caused by such schedules are “obviously not ideal … so you’ve got a patient who should be in isolation who fits the case definition for viral haemorrhagic fever and they’re not getting the result for two weeks” (WHO clinical staff, Freetown):

*“So*, *[samples] should both be tested simultaneously*. *Often that doesn’t happen*, *specimens get stuck at Kenema*. *They’re tested for Lassa and then they spend two weeks sitting there because there’s no transport for them to go to Bo which is 45 minutes away to get a test for EVD*.*”*

(WHO laboratory staff, Freetown)

### Ownership of diagnostic results

Alongside these longstanding issues in the specimen transport and referral system, there are issues of data ownership and reporting that further exacerbate diagnostic delay, even where local lab capacity was not the bottleneck:

*“When we are seeing the IDSR reports week on week*, *we are seeing them [CPHRL] report an EVD result*, *but no Lassa results which they should be able to give (and also Marburg)*, *and what we are seeing from Kenema is Lassa results but no EVD results”*.

(WHO clinical staff, Freetown)

There was a perception from multiple respondents that there was a lot of ‘politics’ within Sierra Leone’s public health laboratory system that affected which labs had the credibility or authority to run and report diagnostics for different conditions. These politics were even perceived by one key informant as affecting central MOHS staff’s ability to access the LF laboratory, implying they were prevented from entering because of partner involvement with the facility, as well as the timely receipt of results:

*“We were there*, *WHO with the Ministry and CDC*, *so why can’t a Ministry person enter a Ministry lab*? *So it seems to be a little bit outside of the whole [system] … and they [MOHS] don’t… have access to the Lassa lab results”*

(WHO laboratory staff, Freetown).

Respondents viewed those politics as emanating from either the need to control data for future research or publication opportunities, or from the influence held by some external partners within Sierra Leone’s fragmented health system [37].

For example, the IDSR Guidelines identify Kenema as the reference lab for LF diagnostics. Therefore, other sites did not report LF results, even though they are now permitted to conduct the relevant tests, with implications for the agility of the overall system.

### Impact of delays on patient management and outcomes

As acknowledged by the Sierra Leone MOHS in the National Health Laboratory Strategic Plan, all of these limitations affect patient management and therefore outcomes [38], and reduce health worker motivation to send samples from febrile patients for further diagnosis.

*“That disconnect between results coming through in a timely fashion actually means that the clinicians may not feel there is value to flagging up*, *whether it’s for meningitis and getting a result which makes a clinical difference to the child”*

(WHO clinical staff, Freetown)

For HCWs at PHUs, delays in receiving results or feedback on patients referred for further diagnostics also has implications for isolation, surveillance, contact tracing and rodent control measures, as well as their own safety. Knowing whether a patient is LF positive enables health workers to be on the look-out for additional cases of the disease:

*“for some patient[s] we will write queries and what we mean…is guess*, *i*.*e*. *we do not know exactly the sick so we send them for correct diagnosing in the big hospital and we expect them also to tell us what is the outcome of their own test result and we will know the exact sickness that patient is suffering from*. *So feedback is really important”*

(HCW, Kenema district, Ebola-/Lassa+ chiefdom)

The IDSR Guidelines state that “health workers (including CHWs) conduct surveillance activities at all levels of the health system so they can detect public health problems of concern to their community” (IDSR Guidelines, p. 30). Receiving a positive result for an outbreak prone disease such as LF therefore helps HCWs engage in precisely the kind of activities that the MOHS expects of them, to maintain a functional surveillance and outreach system:

*“[If] it happened to be a Lassa fever [diagnosis] then we will communicate to the community people that those who escorted the patient might probably get the sick”*

(HCW, Kenema district, Ebola-/Lassa+ chiefdom)

By definition, failing to receive timely feedback on results necessarily undermines HCWs’ ability to take up this work and further weakens disease control efforts.

Health Care Workers are also expected to enhance infection prevention and control (IPC) practice in response to confirmation of outbreak-prone diseases [27]. The Ebola outbreak in Sierra Leone had a marked effect on IPC practice at all levels of the health system [61], partly as a result of HCW fears of contracting the disease, but also concerted sensitisation campaigns by the MOHS and NGOs, and improved access to IPC supplies, including personal protective equipment (PPE):

“*of course previously before Ebola… you will see someone touching a patient with his bare hand without using any glove because of lack of awareness*, *and they do not have the knowledge*, *but when Ebola struck*, *we were made to understand all the necessary precautions*, *even [if] we received a simple case*, *whether be it a fever or not we put on our gloves first before touching the patient—but before we were not doing this*, *so this is one of the change[s] after Ebola*.*”*

(HCW, Kenema district, Ebola+/Lassa- chiefdom)

However, there is some evidence that as time has passed, IPC practices may have reverted to pre-Ebola levels. A PHU facility survey conducted in Kenema in 2016 (towards the end of the Ebola outbreak) and again in 2020 found that the proportion of IPC non-compliant facilities had increased from 4% to 100% [62]. Several HCWs noted this change: *“so everybody [was] doing the hand washing*, *but that has declined now since after Ebola”* (HCW, Kenema district, Ebola-/Lassa+ chiefdom). In a setting where standard precautions or barrier approaches to clinical work may not always be routinely applied, receiving timely information about outbreak-prone pathogens circulating in a PHU’s catchment area could help to encourage HCWs there to protect themselves, or indeed for supplies of PPE and other IPC materials to be surged to the relevant health facilities:

*“Three or four months ago we had a Lassa fever case… but we never knew the patient was having Lassa fever*, *my boss always tell me to dress up properly wearing gloves*, *I just took the risk that day*, *my boss told me that [I should] be wearing gloves all the time*, *but you do careless about it*.*”*

(HCW, Kenema district, Lassa-/Ebola- chiefdom)

This respondent made it clear that barrier IPC was not ordinarily practiced by her in the absence of receiving word of any additional risk, highlighting again the importance of functional, timely diagnostics and feedback loops.

This is particularly true outside of Kenema District, where HCWs are not primed to expect endemic LF cases, generally do not receive any specific training on LF, and therefore may be even less cautious in their IPC practice:

*“I think in Kenema it may be better inculcated into the nursing staff there because they have seen children who have started off as an undifferentiated febrile illness and three days later start bleeding*, *and then they think ‘heck*, *what’s happened to the last three days*?*’*, *and because of that they probably have a better IPC process”*

(WHO clinical staff, Freetown)

Similarly, HCWs from outside Kenema are even less likely to suspect undifferentiated febrile patients of having LF, refer them promptly, or initiate the diagnostic processes needed for confirmation, while systems for getting specimens to Kenema are even slower in non-endemic districts. Kenema also houses the only team in the country trained and equipped to conduct LF surveillance and outreach work once a LF patient is identified, another factor that can delay a timely and systemic public health response.

### A study in delay: Magburaka, Tonkolili 2020

One key informant provided a detailed description of a LF outbreak that occurred in Tonkolili district, in the north of the country in January 2020. This clinical case study is described below to illustrate many of the challenges associated with disease surveillance and response for LF already described above, how they affect the overall functionality of Sierra Leone’s public health response system, and particularly the extent to which they are exacerbated when a case emerges outside of the historical LF region.

Not considered to be in the traditional LF belt, Tonkolili District experienced a small outbreak of LF at the Masanga Hospital, in which two HCWs died in November 2019. Yet in 2020, it took 11 days to diagnose a LF-infected pregnant woman.

On January 24^th^ 2020, a pregnant woman at 22 weeks’ gestation from Yoni Chiefdom in Tonkolilli district, with no history of travel to the traditional LF belt, and no known contact with a Lassa case, presented at a PHU near her home with abdominal pain, diarrhoea and vomiting and fever. She was treated there for a urinary tract infection and discharged a couple of days later. She did not get better, so she went to a traditional healer, and then to another PHU in Yoni Chiefdom, by which time she had deteriorated. On or around 27th January, she was referred by the national ambulance service to Magburaka Government Hospital, and arrived there around 2am on the 29^th^ January. On arrival at Magburaka Government Hospital, she was having a seizure. She had no previous history of epilepsy and had a negative malaria RDT. She also had high blood pressure and protein in her urine and was therefore started on treatment for eclampsia.

Sadly, intrauterine death of the foetus was declared. The midwife called a visiting NGO clinician (who was not in Magburaka at the time, but who had worked extensively in the LF belt as well as in a clinical role during the 2014–2016 Ebola outbreak) to discuss the case. They advised continuation of the eclamptic treatment protocol and induction of intrauterine fetal demise. Over several hours the patient was reviewed and examined by several medical and midwifery/nursing team members. The visiting clinician called to get an update and advised that all notes should be checked for any evidence of fever. On the patient’s referral paper, fever was documented, and the visiting clinician advised to treat as suspected LF. A specimen was drawn by the DHMT and the MOHS was informed, but the patient unfortunately died an hour later.

Once the visiting clinician suspected LF, and before the specimen was drawn, the hospital HCWs increased their use of PPE to include gloves and face shields, and they stopped conducting invasive procedures. Until then, several procedures had been conducted without appropriate PPE, both at the Government Hospital, and at the PHUs visited by the patient since no one had suspected LF.

Some of the HCWs at the hospital who had also worked previously in the LF belt later informed the visiting clinician that they too had suspected LF, but had either not vocalised this concern, or had been advised by more senior colleagues that they must be wrong. Retrospectively it was also found that the patient had been bleeding from her cannula sites and a laceration on her leg.

As indicated in the IDSR and in the Specimen Referral Guidelines, the sample drawn from the patient should have been transported to Kenema District for LF diagnostics, and to Freetown for Ebola diagnostics. However, since the lab at Makeni Government Hospital had historically been able to test for Ebola and LF, the sample was sent there instead, as it was closer to Tonkolili. Due to issues with supply of reagents in Makeni, the sample was untested and was returned to Magburaka, before eventually being transported to the Kenema and Freetown labs. Five days after the sample had been originally drawn (29^th^ January), the clinical team at Magburaka were notified on 2^nd^ February through informal channels that the sample was positive for LF. On 3^rd^ February they were notified that it had been confirmed positive in Freetown, and it was only on Tuesday 4^th^ February, 11 days after the patient first presented at a PHU, that official confirmation was received from the Kenema lab.

Positively, and despite this delay in confirmation, the DHMT had started the process of contact tracing and line listing as soon as the informal confirmation was received, rather than waiting another 48 hours to receive official notification. Later, the Kenema LF outreach team travelled to Tonkolili to support this process with their specialised skills and training. The patient’s body was released to the patient’s family prior to receiving case confirmation, since the hospital morgue was unable to maintain appropriately cool temperatures, and there were concerns about causing unnecessary distress to relatives if it were retained in those conditions. The DHMT then supported the family in conducting a safe and dignified burial.

While Magburaka Government Hospital, Tonkolili is not in the traditional LF belt, the outbreak at the neighbouring Masanga Hospital three months earlier might have been expected to presage a lower threshold for suspecting LF; similarly, when the patient began displaying haemorrhagic symptoms, a suspect VHF alert should have been raised. The events reported here are also not the first time that LF has been identified in Tonkolili, with at least two other studies reporting the disease in that district [6,24]

Instead, suspect case identification was delayed, in part because the assumption of most HCWs was one of LF negativity, rather than the inverse, despite the patient’s atypical presentation. It was also not recognised that pregnant women often act as sentinel cases for a new VHF outbreak due to their worse outcomes [16] and better engagement with the health system. As a result, the patient’s referral to higher levels of the health system was also delayed, potentially contributing to the poor outcome. Meanwhile, several HCWs across multiple sites were exposed, and conducted invasive procedures without appropriate PPE. Given that maternity is a specialty with risk of exposure to body fluids and sharps injury, the risk of nosocomial infection was particularly high. Finally, the delays in case confirmation as a result of various challenges in the laboratory system not only delayed initiation of surveillance activities, but had the patient survived, she would have been kept in isolation awaiting results of a diagnosis for much longer than necessary before initiating appropriate and timely treatment for LF. These delays would also have affected clinical outcomes had the result been negative, since the patient may have needed post-abortion care, and this would not have been available to her while on a VHF isolation ward.

### Setting the detection threshold

This case study of a LF outbreak in a district outside the traditional LF belt also raises questions about appropriate risk thresholds for case detection for outbreak prone diseases in Sierra Leone. The Tonkolili case study and the data reported in this study from HCWs and public health workers suggest that alongside reduced access to confirmatory diagnostics (both inside and outside Kenema), existing case definitions set too high a threshold for detecting LF, and that this may be contributing to a reduction in identified LF cases:

*“The bottom line is that too few patients are being identified as possible Lassa, probable Lassa, having their serology, having their PCRs taken, being put into the system”*

(MOHS clinical staff, Freetown)

However, lowering this threshold, while likely to improve LF case detection, would require substantial resources to be redirected to a disease that likely contributes little to the overall burden of morbidity and mortality in Sierra Leone. In an extremely resource limited context where other febrile diseases dominate, the benefits of doing so would need to be carefully weighed against the costs associated with re-orienting parts of the health system towards better identification of LF, particularly given the volume of undifferentiated febrile illness. As one infectious disease clinician noted, *“I think if you’ve put everybody with an undifferentiated fever in IDU [infectious disease unit] you would have a very full IDU”* (Infectious Disease Clinician, Freetown).

Actors in the MOHS are aware of these tensions between the need for good VHF surveillance and the costs of improving the system. Rolling out a more sensitive case definition nationally and down to the PHU level would be desirable from a disease control perspective, especially given the conflation of all VHFs within the IDSR, but would inevitably divert resources away from other MOHS activities, and may not even be possible, because of laboratory capacity and supply chains:

“*If not*, *[it would be] because of the cost involved*, *everything has a cost*, *but other than that that is the best weapon*. *And not only for Lassa now*, *but since we have the Ebola virus in circulation*, *we have Marburg also hanging somewhere*, *so for us to be on the safer side*, *that would be the one move that would keep us safe*. *That every case that comes to the facility you test for malaria and it’s negative*, *and they still run a fever*: *don’t try other antibiotics*, *test*, *go and do the screening because for now we are testing for four or five viruses*, *if you take the sample to the lab they will test for four or five viruses and see which one we are playing with*. *That would be the safer*, *the better way of doing it*. *[But] I don’t know whether we would have the reagents for the testing”*

(MOHS surveillance, Freetown)

Not only would such a strategy increase demand on laboratories because each suspected VHF case would need to be screened against multiple viruses, but it would also substantially increase the number of samples taken and transported for diagnostics:

*“The challenge is that if you want to manage, if you want to identify your case as Lassa you have to have a broad case definition, but you are going to be testing a lot of people.”*

(WHO clinical staff, Freetown)

It is also not clear from the scientific literature exactly how infectious LF is, in terms of human-to-human transmission either in the community or in health care facilities, and examples like the case at Magburaka Government Hospital raise queries for clinicians:

*“I mean I think the big questions for me that came up from this case*, *were you know*, *would this patient have just passed through this maternity department*, *died*, *body gone back to the family*, *and nobody would ever have been any of the wiser*. *And would it have mattered*? *Because nobody*, *as far as we know*, *nobody got infected from this patient*, *even though they weren’t following a higher level of PPE*”

(NGO clinician, UK)

The lack of HCW infection in this one case does not mean that LF risk mitigation should halt altogether, but it does draw attention to the way that inherent fragilities in the system may lead clinicians to reduce their risk perception; if HCWs are not resourced to manage outbreak prone diseases appropriately, but there are not wide-reaching consequences from this, their risk perception for subsequent exposures to suspected VHFs may reduce, thus placing them at greater risk.

It is also worth noting that alongside these systemic challenges associated with the case definition and diagnostics, health worker attitudes and health system culture may also be contributing to this low detection rate:

*“There is a, there’s a kind of a hope that it isn’t and therefore people tend to send off the tests when it clearly is [positive], which means that the proportion of positives is far higher than I would expect.”*

(MOHS clinical staff, Freetown)

Therefore, if there is a desire to improve LF surveillance, alongside resolving systemic issues, it will also be necessary to determine how to tackle a culture that does not always favour infectious disease notification, or dissuades HCWs from referring suspected case to the appropriate facility:

*“I don’t know if it’s because they’re frightened of the implications of saying that you’ve got a viral haemorrhagic fever in your hospital”*

(NGO Clinician, UK).

The Magburaka case study highlights the ways in which both formal and informal components of the health system can lead to delays in LF (and by extension, other febrile illness) case identification and therefore worse outcomes for patients, health workers, and the overall system. However, alongside raising queries about *how* systemic improvements might be brought about, given the non-specific nature of many prevalent diseases and health system resource limitations, it also raises critical questions about how much more sensitivity *should* be introduced into Sierra Leone’s public health surveillance and response system, given other constraints, competing demands, and population health risks.

## Discussion

By utilising an in-depth case study approach, this empirical study has found that there are several challenges to the timely identification, diagnosis, and referral of suspected LF cases in Sierra Leone, including: a lack of specificity in the LF case definition; a tendency at PHUs to diagnose diseases through serial testing or treatment for more common febrile conditions (adding delays to the wider process of narrowing a differential diagnosis); specimen transport resourcing limitations; inflexibility of specimen transport organisation; fragmentation in the public health laboratory system; and inefficiencies in laboratory reporting processes that affect timely diagnostics and feedback for clinicians. These health system challenges are not unique to LF, and have implications for the effectiveness of the wider IDSR system in Sierra Leone, patient and outbreak management and response. Identification of ways to improve the system requires consideration of appropriate risk thresholds for surveillance of specific diseases, and how to balance those against other population health needs, particularly where resources are scarce. Therefore, health system challenges to early identification of LF *specifically* have implications for the effectiveness of the wider IDSR system in Sierra Leone more *generally*.

This study has also demonstrated that there are several barriers to alleviating these identified challenges. First, consideration of ways to improve only LF surveillance exemplifies the tension between the Global Health Security Agenda, and true progression towards Universal Health Coverage (UHC), by forcing a choice between two apparently conflicting approaches to improving population health. Many have argued [63,64] that the solution is generic health system strengthening and improved synergies between the two agendas. Improving UHC in Sierra Leone, particularly in terms of quality of care at the primary healthcare level (identified by the MOHS as *the* critical area of focus for improving health outcomes [38]), might encourage earlier care-seeking, and facilitate appropriate and timely referral from PHUs that would support better LF surveillance and response as a by-product. However, this approach would take substantial resources [65], and is unlikely to yield improvements to the surveillance system in the short term.

Second, studies have shown that most LF cases are rodent-initiated [8], and in Sierra Leone rarely transform into generalised human-to-human outbreaks. As such, it is hard to argue for the diversion of limited resources away from primary care and towards improving the above surveillance ‘building blocks’ for a specific vertical disease programme. Similarly, HCWs’ use of malaria treatment in the diagnostic process is rational in this high-prevalence setting where the verticality of the donor-funded malaria programme means this treatment is well supplied [66], while other diagnostics are not readily available. Expanding access to lab diagnostics for all febrile illness would come at considerable cost and complexity, although resources-willing, may be justifiable in a setting like Sierra Leone.

Developing a more sensitive, stratified LF case definition and strengthening the associated diagnostic processes and pathways in the ‘traditional’ LF belt of southern Sierra Leone would provide opportunities for targeted improvements that would not require a wholesale health system reorientation towards a potentially low-prevalence disease. However, this approach is also not straightforward: it would require regular training and awareness-raising of HCWs operating at all health system levels in that region. Ideally, a high performing, validated RDT for LF could be added to the routine use of malaria RDTs’ at the primary healthcare level, facilitating rapid and early detection of LF cases, as well as more rapid differential diagnostic processes for other common febrile conditions. However, those RDTs are not yet available [18], and given the limited nature of accurate data on LF’s current epidemiological status and geographical spread within Sierra Leone [9], alongside the wider fragilities in the system that have implications for identification of other febrile diseases, this verticalised approach, even in the so-called endemic areas, risks further fragmenting the system.

Improving LF surveillance in the traditional LF belt alone would also risk undermining recent efforts in Sierra Leone towards an integrated and universal disease surveillance and response system that would benefit from wholesale strengthening [67]. Further, determining the best approach to sentinel surveillance for VHFs in a country with such high malaria prevalence and likely high rates of malaria/VHF coinfection is challenging, particularly if recommendations are realistic about resource limitations.

This study therefore recommends a hybrid approach to LF surveillance with a focus on improving LF surveillance ‘building blocks’ both in the traditional LF belt districts, and simultaneously at maternity wards throughout Sierra Leone. Evidence shows that maternity patients often play a sentinel role in helping to detect VHF outbreaks [68–72], and there are additional complexities for case management and IPC (through the additional risk of nosocomial transmission) with pregnant VHF patients [73]. Therefore, focusing improvements to VHF surveillance system components and processes to a) the traditional LF belt, and b) national maternity units could offer the appropriate balance for more targeted surveillance that reduces the risk of the worst patient outcomes, without over-emphasising a relatively low prevalence disease. This would not only improve clinical identification of LF cases, but would also reduce the epidemiological knowledge gap on LF in Sierra Leone. Further, strengthening VHF surveillance at maternity units (with a re-worked case definition specific to maternity cases, and more training of health workers at those sites) would likely result in downstream benefits for the wider national IDSR system, and for the health system.

COVID-19 now presents as yet another non-specific febrile illness within Sierra Leone’s disease ecosystem which, like Ebola before it, is negatively affecting health service delivery and utilisation [74]. Marburg has, for the first time in West Africa, been detected in humans in West Africa [60]. There is therefore even more of an onus to resolve some of the practical challenges to disease surveillance and response throughout the country. Improvements to the surveillance and response systems for febrile illness would have generalised benefits for COVID-19 response, and improving LF case identification, diagnosis, and therefore case management and surveillance would be one small step to speeding up the differential diagnosis process for other febrile conditions that present in similar ways, and therefore to improving health outcomes in Sierra Leone more generally. In this way, steps might be taken locally towards generalised health system strengthening by leveraging the political will and capital emanating from the Global Health Security Agenda.

### Study limitations

The qualitative nature of this study means that its findings may not be generalisable beyond Sierra Leone. It also reflects the specific views and perspectives of respondents, since they did not constitute a random or representative sample of HCWs or of those working at a policy level within the health system, and should not be interpreted as reflective of the views of health system/policy actors whose perspectives were not included in this research. However, the findings reported here provide rich insights into the structures, systems, and mechanisms affecting Sierra Leone’s infectious disease surveillance and response system; help understand implementation gaps in that system; and can help draw attention to health system components that may affect the functionality of surveillance and response in other comparable settings.

Recruitment of respondents (both HCWs and key informants) via gatekeepers may have introduced biases such as the deliberate selection of participants perceived likely to voice views that are favourable to those helping select them. Further, CHWs and HCWs may have perceived the interviews and FGDs as opportunities to voice grievances in the hope that their complaints might be addressed. The validity of these data were assessed by triangulating data against one another, as well as against the content of policy/guidance documents, and the wider literature on Sierra Leone’s health system.

## Supporting information

S1 Table Policy documents included in review. (DOCX)

S1 File Key informant interview example topic guide. (DOCX)
