# Supplementary material for: Beyond Lassa Fever: Systemic and structural barriers to disease detection and response in Sierra Leone
Source: PLoS Negl Trop Dis. 2022 May 19;16(5):e0010423. doi: 10.1371/journal.pntd.0010423 (PMC9159599; doi:10.1371/journal.pntd.0010423)
Supplement: S1 Table — (DOCX) [file pntd.0010423.s001.docx]

Table 1 - Documents included in review

|  | **Documents Included in Review** |
| --- | --- |
| 1  2  3  4  5  6  7  8  9  10  11  12 | Ministry of Health and Sanitation. Technical Guidelines for Integrated Disease Surveillance and Response. Freetown, Sierra Leone; 2015.  Ministry of Health and Sanitation. National Rapid Assessment of Laboratory Capacity and Systems. Freetown, Sierra Leone: Government of Sierra Leone; 2015.  Ministry of Health and Sanitation. National Community Health Worker Strategy 2016-2022 (Draft). Freetown, Sierra Leone: Government of Sierra Leone; 2016.  Ministry of Health and Sanitation. Sierra Leone National Medical Laboratory Strategic Plan 2016-2020. Freetown, Sierra Leone: Government of Sierra Leone; 2016.  Ministry of Health and Sanitation. National Community Health Worker Policy 2016-2020. Freetown, Sierra Leone: Government of Sierra Leone; 2016.  Ministry of Health and Sanitation. National Health Sector Strategic Plan 2017-2022. Freetown, Sierra Leone: Government of Sierra Leone; 2017.  Ministry of Health and Sanitation. Special Pathogen Referral, Testing and Reporting Algorithm. Freetown, Sierra Leone; 2018.  Ministry of Health and Sanitation. National Action Plan for Health Security 2018-2022. Freetown, Sierra Leone: Government of Sierra Leone; 2018.  Ministry of Health and Sanitation. DRAFT: National Guidelines for Integrated Laboratory Specimen Referral. In: MOHS, editor. Freetown, Sierra Leone2019.  Ministry of Health and Sanitation and WHO. Standard Case Definitions of Priority Diseases, Conditions, and Events for Health workers in Sierra Leone: A clinician’s handbook, Feb 2016  Ministry of Health and Sanitation and WHO. Participant Training Manual: Clinician’s Role in Disease Surveillance and Response, Updated July 2017  Ministry of Health and Sanitation All Hazards: Public Health Incident and Emergency Response Plan and Emergency Preparedness, Resilience and Response Concept of Operations |
