# Supplementary material for: Beyond Lassa Fever: Systemic and structural barriers to disease detection and response in Sierra Leone
Source: PLoS Negl Trop Dis. 2022 May 19;16(5):e0010423. doi: 10.1371/journal.pntd.0010423 (PMC9159599; doi:10.1371/journal.pntd.0010423)
Supplement: S1 File — (DOCX) [file pntd.0010423.s002.docx]

## Example Key Informant Interview Topic Guide

1. Please could you tell me a bit about your role here at the _____?
2. Please could you tell me about the role of the _____ within Sierra Leone’s public health and surveillance system?
3. Please could you describe the way that Lassa fever surveillance is integrated within the _____?
4. Are there local SOPs or other guidance documents for Lassa fever surveillance, diagnostics, case management etc? What are these?
5. What challenges does the ____ face with integration?
6. How does the Lassa fever case identification/diagnostic system work?
7. What is the threshold for triggering a public health/RRT response for Lassa fever?
8. How does it fit within VHF surveillance more broadly? Do you think this is the right approach?
9. (if no) what changes would you make to the system?
10. What barriers or opportunities to making those changes?
11. What is your view of the case definition for Lassa fever in SL, and its impact on Lassa surveillance? Is it easy to implement?
12. How is information flow for Lassa managed from a ____ perspective?
13. Are there any partners working on Lassa fever?
14. What is your view of their involvement with Lassa fever?
15. How often do _____ receive alerts for Lassa fever? From districts other than Kenema?
16. Since you’ve been in role, have there been any Lassa/query VHF case investigations? If yes, could you talk me through your experience with one of those cases?
17. Should a suspect Lassa case be isolated until diagnosis is confirmed? Are the facilities available to support that?
18. Is Lassa fever treatment free? How are supplies for Lassa fever treatment integrated within national supply chains?
19. Should patients diagnosed with Lassa fever receive free treatment for other comorbidities or coinfections? How does that work in practice? Are health workers aware of the protocols?
20. What critical changes would you make – if any – to the Lassa/VHF surveillance and diagnostic systems?
21. Is there anything I haven’t asked about that you think is critical to understanding the organisation of the ______ and its management and response to Lassa fever?
